# Lord Lister: IX. The Drainage Tube, and Ligatures

**Published:** 1918-03-23

**Authors:** 


(*To be continued.*)

### LORD LISTER.

### IX.—The Drainage Tube, and Ligatures.

In order to allow the free escape of the discharge from the interior of the wound, Lister designed the drainage tube. He was not the first to design it. It had been used years before by Chassaignac; but Lister did not know of Chassaignac's device, and invented it afresh. The first occasion on which he used it was a memorable one, for the patient was no less a person than Queen Victoria herself. Lister had opened a deep-seated abscess in the axilla, and had inserted, for the purpose of drainage, a strip of lint soaked in carbolic oil. When, however, in dressing the wound, he withdrew the lint, the withdrawal was followed by a gush of pus, showing that the lint had acted as a plug rather than as a drain; so he took the rubber tube from the spray producer that he had used to produce local anæsthesia at the operation, cut holes in it, attached ligatures to one end, inserted the other end into the wound, and so improvised a drainage tube which he left to soak all night in carbolic acid, and pushed next morning into the depth of the wound. At the next dressing he was gratified to find that instead of a gush of thick pus, as on previous occasions, the withdrawal of the drain was followed by only a few drops of clear serum. This was in 1871, and thereafter the use of rubber drainage tubes soon became a practice of routine, both for those who adopted Lister's system in other respects, and for those who did not.

As long as strong solutions of carbolic acid were employed to cleanse the surfaces of the wound, discharge from these surfaces, though no longer purulent, still occurred in considerable quantity, and necessitated the continued employment of drainage tubes. As weaker and weaker antiseptics were used to cleanse the surfaces of the wound, the precautions necessary to secure free drainage became less and less necessary, until now no antiseptic at all is employed, wounds are habitually completely closed on the operation table. Serum still collects in the interior of the wound in some cases, and necessitates reopening of the wound to let it out, but this is exceptional, and Lister's ideal of dispensing altogether with drainage, though not yet realised universally, is so in a great many cases of operation. When drainage is needed nowadays, it is needed to allow the escape of a small quantity of thin or almost clear inodorous serum, instead of the deluge of thick stinking pus that flowed from operation wounds in pre-Listerian days.

Even after the discharge had been reduced to a minimum, there remained the difficulty of the ligatures. As already described, one or both ends of the ligatures of flax, cotton, or more usually soft silk, was left long, and all the ligatures employed were collected in a bundle and led out of the wound at the same place. Under the system that prevailed before Lister, the ligatures were regarded rather as an advantage than otherwise to the subsequent course of the wound, for it would happen very often that the edges of the skin would unite, while the internal surfaces secreted large quantities of pus. The result was that the cavity of the wound became practically an abscess cavity, with the pain, the rise of temperature, and the constitutional disturbance that attend an unopened abscess. The issue of the bundle of ligatures from the corner of the wound prevented the edges of the skin from uniting at this spot, and was supposed to act as a drain to facilitate the escape of the discharge. As often as not it acted, as the lint in Queen Victoria's axilla acted, as a plug, and the patient did not obtain the advantage of a drain, but he did suffer the disadvantage of the presence of a foreign body in the depths of the wound, a foreign body, moreover, that was always septic. Manifestly, under these conditions it was not to be expected that a wound would heal aseptically, or as Lister called it, antiseptically, and he set himself to obviate the disadvantages of the ligature.

The first precaution was, of course, to render the ligatures "antiseptic," or, as we should say nowadays, to sterilise them; and this Lister effected by steeping them for some hours before use in a strong solution of carbolic acid. This did put a stop to the introduction of septic material into the wound by the vehicle of the ligatures, but only at the cost of introducing an irritant caustic, and Lister looked round for some means of abolishing altogether the dangling bundle of ligatures. While he was thus engaged, two methods of occluding arteries in wounds and stopping the flow of blood from them were devised, and came into limited use. The first of these was acupressure, a method devised by

☛ Previous articles appeared Jan. 26, Feb. 2, 9, 16, 23, March 2, 9 and 16, p. 503

**LORD LISTER**—(*continued*).

Sir J. Y. Simpson, whereby a steel needle was passed through the tissues on both sides of the artery in such a way as to exert upon the artery sufficient pressure to stop the flow of blood. It was not necessary that the needle should enter the wound at all. It might be completely buried in the tissues. It could be easily sterilised, it could be allowed to remain as long as was needful, and it could be withdrawn at will when it was supposed that the occlusion of the artery was completed by the adhesion of its walls. It is manifest that if only the method could be depended upon to arrest the hæmorrhage permanently, its advantages were very great; but the internal coat of an artery shows little tendency to cohere, even when the sides of the artery are pressed strongly together for many hours, and therefore there is always danger of hæmorrhage when the needle is withdrawn. Simpson advocated the method with great enthusiasm, but he gained but few adherents. The method was never widely utilised, and soon fell altogether into disuse, probably for the reason indicated. It does not appear that it was ever tried by Lister.

The other method of occluding arteries and arresting hæmorrhage was known as torsion. The end of the artery was seized in a pair of flat-bladed forceps, which were then twisted round several times on an axis coinciding with that of the artery itself. The effect was to tear through the inner coat of the artery, which then curled inwards and afforded a rough edge which provoked coagulation of the blood, while at the same time the middle and outer coats were blended into an indistinguishable mass. Torsion also had its enthusiastic advocates, and cases were published in which it had been employed without the occurrence of secondary hæmorrhage for the occlusion of large arteries. It is still employed to stop the oozing from small vessels, but is not now employed for spouting arteries. It could scarcely give the restful certainty of permanent occlusion that is afforded by the employment of the ligature; and whatever its merits, it never came into general use as a substitute for the ligature; nor does it appear that it was ever employed by Lister.

He was, of course, familiar with the fact that foreign bodies, such as bullets, needles, and fragments of glass, might become encapsuled in the tissues and remain for years without exciting suppuration, and he saw at once the reason why they might do so and yet the ligature round an artery never did so. In his own words, "Unlike the glass or metal, the thread is porous, and contains in its interstices putrefactive germs, which, developing in the serum that bathes the ligature, give rise to the acrid products of decomposition, and these, in their turn, stimulate the surrounding tissues to granulation and suppuration. If, however, the thread were steeped in some liquid calculated to destroy the life of the germs in its interstices, and the wound by which it was introduced were treated antiseptically, the ends of the ligature being cut short, it might be left with confidence that its presence would not interfere with primary union, or occasion any disorder in the surrounding parts." At first, therefore, his utmost ambition with respect to the ligature was that it might lie like a bullet or a needle, as an innocuous but permanent foreign body in the tissues; but he soon saw reason to believe that he could do much better than this.

While he was ruminating over the problem presented by the retention of ligatures in the wound, and the inevitable delay in healing that their presence necessitated, a momentous experience happened to him. He was treating antiseptically a wound in which a portion of the tibia was exposed, deprived of periosteum, and was manifestly dead. He was waiting for the dead portion to exfoliate and become separated from the living portion, so that he could remove the former, now become a foreign body in the wound, and so remove an obstacle to the healing process. One day, however, in dressing the wound, he saw to his surprise that instead of presenting the dead white opacity of necrosed bone, the exposed portion of the tibia exhibited a delicate blush of pink, clearly indicating that the granulations beneath had penetrated so near to the surface of the bone that their colour showed through. On trying to pass the flat end of a probe between the outer surface of the bone and the granulation that enveloped it, he found that the probe would penetrate but a very little way, beyond which the granulations adhered to the bone. The inference was clear. The granulations were eating the bone away. They were absorbing and removing its substance. The mere fact that dead bone separates from living bone and comes away as a sequestrum might have taught him the same lesson. However, the lesson was now learnt—the lesson that a foreign body in a wound, if organic in nature, can be absorbed and removed by the granulations of the wound. But if bone is an organic substance, so is silk, and if bone can be thus absorbed and removed by the granulations, why should not silk also be absorbed and removed, providing that in the one case as in the other "decomposition," that is to say, suppuration, can be prevented? The idea was an inspiration, and Lister proceeded at once, after his custom, to make experiments to test its truth.

(*To be continued.*)

